# Inflammation, mental health, and alcohol behaviors: Testing links leveraging a familial community sample

**DOI:** 10.1016/j.bbih.2026.101229

**Published:** 2026-03-26

**Authors:** Ryan Bruellman, Chandra A. Reynolds, Andrew Smolen, Donald Evans, Daniel E. Gustavson, Jarrod M. Ellingson

**Affiliations:** a Genetics, Genomics and Bioinformatics, University of California Riverside, Riverside, CA, United States; b Department of Psychology, University of California Riverside, Riverside, CA, United States; c Institute for Behavioral Genetics, University of Colorado, Boulder, CO, United States; d Department of Psychology and Neuroscience, University of Colorado, Boulder, CO, United States; e Department of Psychiatry, Anschutz Medical Campus, University of Colorado, Denver, CO, United States

**Keywords:** Inflammation, Alcohol, Depression, Twins

## Abstract

Evidence that inflammation is related to poorer mental health largely comes from clinical samples. This pre-registered study examined whether these findings extend to depression, alcohol use, and alcohol use disorder (AUD) in a large sample of community adults (N = 972, *M=*33.4 years, range = 28-49), including a subset of participants from same-sex twin pairs. Analyses primarily examined C-reactive protein (CRP) and a pro-inflammatory cytokine index of three cytokines: interleukin(IL)-1β, IL-6, and tumor necrosis factor alpha (TNF-⍺). This pro-inflammatory index averaged the three cytokines after each was placed on a standard-normal distribution (*M* = 0[SD = 1]). Covariates were age, sex, diet relevant measures, and anti-depressant use. Further, we pre-registered analyses to follow up significant effects in the full sample with co-twin control analyses, which compare twins to each other to control confounds that make twins similar. Depression, alcohol use, and AUD were unrelated to CRP and the pro-inflammatory index. Notably, AUD was associated with *lower* levels of four individual cytokines (after multiple-testing correction): IL-1β, IL-4, IL-10, and IL-12. In co-twin control analyses, however, these negative associations were nonsignificant, suggesting familial confounders explain these associations. These findings suggest that associations between cytokines and indices of depression and alcohol behavior may not extend from clinical to community samples. Further, although this study is the first that applies the co-twin control approach to rigorously test the link between inflammation and alcohol behavior, it largely converges with studies that suggest confounders explain the putative protective effect of alcohol behaviors on inflammatory markers of health.

## Highlights

• Pre-registered study (N=972) tested if clinical links between inflammation, depression, and alcohol behavior generalize.
• Co-twin control analyses of same-sex pairs account for all genetic and environmental confounders shared by twins.
• Depression, alcohol use, and alcohol use disorder were unrelated to CRP and a pro-inflammatory index (IL-1β, IL-6, TNF-⍺).
• Higher alcohol use disorder was associated with lower levels of IL-1β, IL-4, IL-10, and IL-12 in the full sample.
• Co-twin analyses suggest confounders explain the putative protective effect of alcohol behaviors on inflammatory markers.

## 1 Introduction

Inflammation is part of the innate immune response against threats, such as infection and injury. Cytokines are a key part of this immune response (Bauer and Teixeira, 2019; Iwasaki and Medzhitov, 2015) and have attracted interest due to their associations with psychiatric symptoms (Liu, 2017). While cytokines may directly affect psychiatric symptoms, or behaviors related to psychiatric disorders may affect cytokines, findings primarily come from clinical samples and may not extend to otherwise healthy adults with low levels of inflammation. The current study aims to advance the literature on the relationship between cytokines and mental health by examining associations with depression and alcohol behavior in a sample of healthy adults.

Inflammatory cytokines have been widely implicated as a biomarker for psychiatric disorders. Seminal work linking inflammation to mental health came from studies showing that about 40% of patients receiving pro-inflammatory Hepatitis C treatments develop major depressive disorder (MDD) symptoms (Dieperink et al., 2000). Meta-analytic work has found similar associations between circulating cytokine levels and depression severity among patients with depression (Dowlati et al., 2010). Additionally, pro-inflammatory endotoxin (lipopolysaccharide) administered in a laboratory setting is associated with greater acute increases in anhedonia and a stronger inflammatory response among depressed participants with elevated inflammation (i.e., high C-reactive protein [CRP] (Savitz et al., 2025) vs. those with low baseline CRP). Whether inflammatory cytokines differentiate depression status in otherwise healthy samples, with generally low levels of inflammation, is unclear. Further, elevated cytokine levels are associated with health problems (e.g., cardiovascular disease; Ferrucci and Fabbri, 2018) that may cause depression (e.g., Bair et al., 2003) or share risk factors with depression (e.g. sedentary behavior) (Xu et al., 2020).

One behavior thought to increase circulating cytokines is chronic, heavy drinking that is characteristic of alcohol use disorder (AUD). Notably, AUD is highly comorbid with MDD (Grant et al., 2015), and elevated cytokines may be a mechanism for their co-occurrence. Inflammation is associated with AUD, regardless of whether patients also have cirrhosis or an infection (Achur et al., 2010; Hillemacher et al., 2018). It is unclear how inflammation is related to alcohol behavior in community samples, but some evidence suggests a curvilinear relationship. Specifically, adults with moderate alcohol consumption (e.g., less than two drinks daily) may have lower cytokine levels (Imhof et al., 2004), but such effects are often negligible when excluding alcohol abstainers or participants with health problems, suggesting possible confounding (Kember et al., 2024; Miller, 2024; Zhao et al., 2023).

The current study examined the relationship between circulating cytokines among healthy adults (*M-*age = 33.4 years old), including twin and non-twin participants. Specifically, we pre-registered analyses (https://osf.io/x4tr3/) to test two research questions: 1) Are heavier alcohol use and AUD symptoms associated with higher cytokine levels?; and 2) Are higher cytokine levels associated with having more MDD symptoms? We hypothesized significant effects for both questions, which would raise the possibility of cytokines mediating the relationship between heavy drinking/AUD and MDD.

## 2 Materials and methods

### 2.1 Participants

Participants were from the first wave of the Colorado Adoption/Twin Study of Lifespan behavioral development and cognitive aging (CATSLife1), recruited between 2015 and 2021 (Wadsworth et al., 2019). All research was conducted according to APA ethical standards, and the Institutional Review Boards at the University of Colorado, Boulder and the University of California, Riverside approved the protocol. Participants provided written informed consent. The CATSLife sample includes a sample of adopted and non-adopted siblings from the Colorado Adoption Project (CAP; n = 597) and a sample of same-sex twin pairs from the Longitudinal Twin Study (LTS; n = 730) (total n = 1327). Participants' ages at the time of blood collection represented established adulthood (*M*-age = 33.41 years, SD = 5.01, range = 28-49). 92.6% of participants identified as non-Hispanic and 7.2% identified as Hispanic.

The following exclusion criteria led to the removal of 355 individuals and included: not completing a blood draw (e.g., due to COVID-19 pandemic restrictions; n = 195), >30 days between completing the blood draw and other study measures (n = 74), current pregnancy (n = 19), and taking medications that affect cytokines (Lisboa et al., 2017; Younger et al., 2014; Zidek et al., 2009) (n = 67). Thus, data from 972 participants were analyzed.

### 2.2 Measures

**Cytokines.** Biospecimens were collected via blood draw during an in-person study appointment. Plasma was collected by venipuncture into Covidien Monoject™ Coated EDTA Tubes, processed according to manufacturer's protocol, and stored at −80 °C. Each plasma sample was assayed in triplicate using a multiplex of pro- and anti-inflammatory cytokines (Quanterix Human Cytokine 10 Plex Array) or C-reactive protein (CRP; R&D Systems Quantikine QuicKit ELISA). Analyses focused on CRP and a pro-inflammatory index averaging interleukin(IL)-1β, IL-6, and tumor necrosis factor alpha (TNF-⍺) after a standard-normal transformation. This pre-registered analytic decision was made based on high correlations observed in prior samples by our group and to avoid an overly stringent significance threshold (i.e., due to correction for multiple testing). Indeed, IL-1β, IL-6, and TNF-⍺ were moderately-to-strongly correlated in the current sample (*r* = 0.54 - 0.71; internal consistency = 0.82). However, for completeness, results for all assayed cytokines are presented in a supplement (interferon-gamma, IL-4, IL-5, IL-8, IL-10, IL-12, IL-22), using a Bonferroni correction for significance of 0.05/8 = 0.00625. Each cytokine measure was log-transformed to address skewness.

**Alcohol use.** Alcohol use frequency and quantity (i.e. drinks/drinking day) were assessed based on the following self-report questions in the PhenX toolkit (NIAAA, N.d.): Think specifically about the past 30 days … “on how many days did you drink one or more drinks of an alcoholic beverage?” (range: 0-30) and “on the days that you drank … how many drinks did you usually have each day?”. From these two variables, we computed a measure of total weekly drinks.

**Psychiatric symptoms.** We used the PhenX toolkit assessed for past-year AUD symptoms based on DSM5 criteria (range: 0-11) (NIAAA, N.d.). Further, we used the semi-structured clinical interview, the Diagnostic Interview Schedule, to assess past-year MDD symptoms (range 0-9) (Robins et al., 2000). To address skew, both AUD and MDD symptom counts were log-transformed.

**Covariates.** All analyses controlled for sex (females = 0, males = 1), age, use of medications used to treat depression, and elements of diet if significantly associated with the respective outcome. Fruit/Vegetable, fiber intake, calcium intake, and added sugar intake were measured using the National Cancer Institute five-factor screener from the PhenX repository (NCHS, 2005; NCI, 2005) using participants’ self-reported intake of varieties of foods and beverages (i.e. cereal, cheese, doughnuts, fruit juice, fruit, green leafy/lettuce salad, white potatoes, cooked/dried beans, other vegetables, tomato sauces, salsa, soda, whole grain bread, etc.). Consumption was scaled to indicate daily frequency: never = 0, 1-3x/month = 0.067; 1-2x/week = 0.214; 3-4x/week = 0.5; 5-6x/week = 0.786; 1x/day = 1; 2x/day = 2; 3x/day = 3; 4x/day = 4; 5+/day = 5. Body mass index (BMI) was measured across all participants by taking their weight (in kilograms) divided by their height (in meters) squared. All variables that were not dichotomized were mean-centered.

### 2.3 Analytic approach

Phenotypic models were conducted using linear mixed-effect (LME) models via the *nlme* package version 3.1-159 in R (Pinheiro et al., 2021). Models using MDD as an outcome and each assayed cytokine measure as a main effect were conducted along with models using each assayed cytokine measure as an outcome and various measures of alcohol use (i.e. frequency, quantity, frequency∗quantity, and AUD) as a main effect. All models were conducted without covariates (unadjusted) and with covariates (adjusted). Sensitivity analyses accounted for race, ethnicity, and batch effects. Additional sensitivity analysis included BMI as a covariate. Due to participant relatedness in our sample, each LME model estimated random effects for the intercept and residual variance by family type (Reynolds et al., 2019). Finally, possible batch effects were accounted for by modeling a random effect for the machine used for cytokine assays.

Analyses included follow-up tests of significant effects using the co-twin control design, which compares twins to each other to control for genetic and environmental confounders that are not part of the putative effect of interest (McGue et al., 2010).

## 3 Results

Descriptive statistics of the analyzed 972 participants are shown on Table 1. Analyzed participants included approximately 48% females and 52% males. Across all participants, fruit/vegetable intake and fiber intake were generally low (Fruit/vegetable M = 2.65 cups/day[SD = 1.30], fiber intake M = 15.36 g/day[SD = 6.92]). Alcohol frequency ranged from 0 to 6 days a week across participants (M = 0.66 days[SD = 0.83]) with alcohol quantity on drinking days ranging from 0 to 12 days (M = 2.00 drinks/drinking day[SD = 1.63]). Participants averaged a low number of symptoms associated with AUD (M = 1.22[SD = 1.84] and MDD(M = 1.16[SD = 2.37]).

**Table 1:** Summary Statistics of Analyzed Sample (n = 972 participants).

	n (% of overall)
Female	469 (48.3)
White	894 (92.0)
non-Hispanic	900 (92.6)
Depression medication use	90 (9.3)

	**Range**	**Mean**	**SD**
Age	28.06 - 48.98	33.41	5.01
Fruit/Vegetable intake (cups/day)	0.25 - 8.50	2.65	1.30
Fiber intake (g/day)	6.21 - 55.34	15.36	6.92
Calcium intake (mg/day)	298.3 - 7024.0	668.70	376.40
Added sugar intake (g/day)	4.03 - 50.14	13.21	6.99
BMI (kg/m^2^)	16.41 - 54.93	25.50	5.85
Alcohol intake (drinks/day)	0.00 - 6.00	0.66	0.83
Alcohol quantity (drinks/drinking day)	0.00 - 12.00	2.00	1.63
AUD symptoms	0.00 - 11.00	1.22	1.84
MDD symptoms	0.00 - 9.00	1.16	2.37

*Notes:* BMI = body mass index, AUD = alcohol use disorder, MDD = major depressive disorder.

**Fig. 1:**
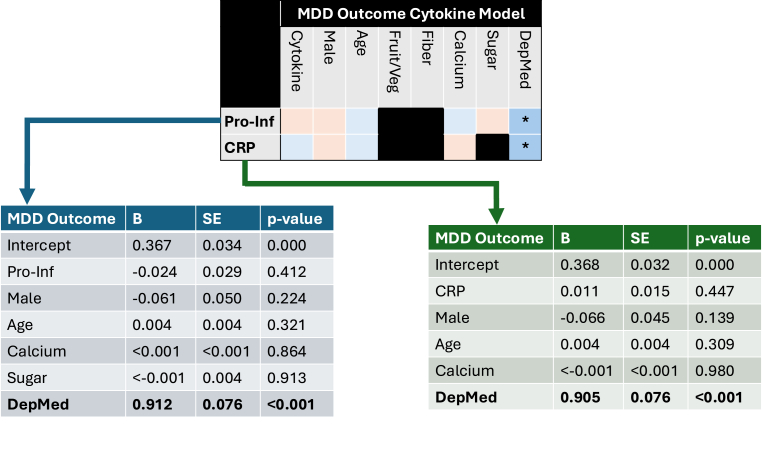
**– LME results for the MDD outcome across pro-inflammatory (Pro-Inf) and CRP main effects**. MDD = Major depressive disorder; Fruit/Veg = Fruit/Vegetable intake; DepMed = Depression medication use; Pro-Inf = pro-inflammatory; CRP = C-reactive protein; B = regression weight; SE = standard error. Blue = positive value; Orange = negative value; ∗ = p < 0.05. (For interpretation of the references to colour in this figure legend, the reader is referred to the Web version of this article.)

MDD was not associated with the pro-inflammatory index or CRP levels (see Fig. 1). The use of depression medication showed significant positive associations with MDD in both models (B = 0.91, p=<0.001). Results of the MDD outcome LME models across all cytokines are shown in Supplemental Fig. 1. Across all models, depression medication showed significant positive associations with the MDD outcome.

**Fig. 2:**
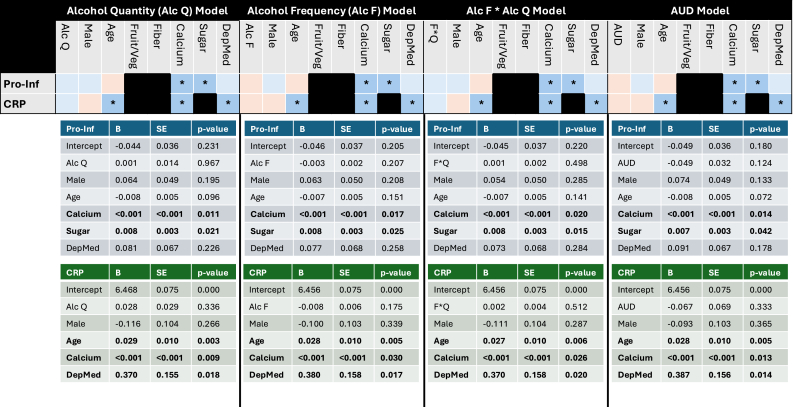
**LME results for the pro-inflammatory and CRP outcomes and alcohol use main effects.** AlcQ = Alcohol quantity; AlcF = Alcohol frequency; F∗Q = Alcohol frequency∗alcohol quantity; AUD = Alcohol use disorder; Fruit/Veg = Fruit/Vegetable intake; DepMed = Depression medication use; Pro-Inf = pro-inflammatory; CRP = C-reactive protein; B = regression weight; SE = standard error. Blue = positive value; Orange = negative value; ∗ = p < 0.05. (For interpretation of the references to colour in this figure legend, the reader is referred to the Web version of this article.)

Alcohol quantity (typical drinks, weekly drinks), frequency, and AUD were not associated with the pro-inflammatory index or CRP (see Fig. 2). Results across all other cytokines were mixed for associations with typical quantity (Supplemental Fig. 2) and weekly drinks (Supplemental Fig. 4). However, there were significant, negative associations for alcohol frequency and AUD with IL-1β and IL-4 when accounting for covariates (Supplemental Figs. 3 and 5). Further, AUD was also associated with lower IL-10 and IL-12 levels after factoring in covariates (Supplemental Fig. 5). Intraclass coefficients (ICCs) from the MDD outcome univariate model with pro-inflammatory and each cytokine outcome univariate model with AUD from the linear mixed-effect results are reported in Supplemental Fig. 6. Monozygotic twins (MZ) pairs showed higher ICCs compared to other family types across 10 of the 13 outcomes. ICCs for IL-6 and IL-22 were comparable between MZ and dizygotic twins (DZ) pairs and DZ pairs had the highest ICCs for IL-5.

These significant, inverse effects were followed up in co-twin control analyses, which account for genetic and environmental confounds that make twins similar. These analyses (Supplemental Fig. 7) suggested that, among twins from the same family, the twin with greater AUD did *not* have proportionally lower circulating cytokines. That is, these negative associations appear to be due to familial confounds that make twins similar rather than a direct effect of AUD on circulating cytokines. IL-1β, for example, showed a nonsignificant but negative between effect (B_AUDBetweenEffect_ = −0.183, p = 0.182). The IL-1β within effect was also negative and nonsignificant (B_AUDWithinEffect_ = −0.231, p = 0.123) with the MZ between effect showing attenuation (B_MZ-AUDWithinEffect_ = 0.159, p = 0.397) indicating possible confounding. Thus, circulating cytokines were largely unrelated to alcohol behavior in this sample of healthy adult twins.

## 4 Discussion

Whereas prior studies have largely focused on the relationship between cytokines and psychiatric outcomes in clinical samples (e.g., diagnosed with AUD or MDD (Dowlati et al., 2010; Leclercq et al., 2012)), the current study examined these associations in a relatively large (n > 900) sample of healthy adults. Contrary to our hypotheses, we found little evidence suggesting cytokines are a biomarker of symptom severity for AUD or MDD in a sample of other otherwise healthy participants in mid-adulthood. Of those effects that were significant, they indicated having more AUD symptoms is associated with lower circulating cytokines, showing possible dysregulated cytokine levels. Accounting for BMI in sensitivity analysis did not alter significance across model outcomes despite BMI having a positive significant association with alcohol quantity, frequency, and AUD related outcomes (Supplemental Figs. 1–5). Follow-up analyses suggested shared familial confounds account for these inverse associations whereby shared genetic and/or familial environmental pathways are salient to alcohol/AUD-cytokine associations rather than direct exposure effects of cytokine. This lack of evidence in our analyses establishing univariate effects did not warrant further consideration of mediation effects in our analysis.

Although our findings may contradict some highly cited work in the extant literature, they align with others. Regarding alcohol use/AUD, we found that the apparent protective effects of heavier alcohol use on inflammation should be interpreted with caution as such associations are likely due to confounds, specifically those familial (i.e., shared by twins). This finding is consistent with those from observational studies that suggest characteristics of abstainers (including those who no longer drink because of poor health) explain the apparent health-promoting effects of alcohol (Sarich et al., 2024; Stockwell et al., 2016). Notably, we conducted additional (non-registered) analyses (reduced *n=*820) excluding alcohol abstainers, which also yielded negligible effects. Regarding MDD, we found no association between circulating cytokines and depressive symptoms. A recent and relatively large (n = 1724) study found that inflammation (CRP) is associated with depression, but this association was entirely explained by confounders (e.g., BMI) (Figueroa-Hall et al., 2022). Our results showed no significances of BMI as a covariate in our MDD-cytokine analyses (Supplemental Fig. 1). These mixed findings on the inflammation-MDD relationship are particularly relevant to recent work suggesting that there may be an “inflammatory” or “immune-metabolic” subtype of MDD (Miller, 2025; Penninx et al., 2025). Thus, it is possible that inflammation may explain specific configurations of MDD symptoms or that immune-related etiology may underlie only a portion of depression cases. Studies with rich, longitudinal data and large sample sizes will likely be particularly useful for better understanding the inflammation-MDD relationship.

These findings also should be considered with respect to characteristics of this study, relative to others in the literature. First, it should be noted that IL-4 and IL-10 are considered anti-inflammatory (Al-Qahtani et al., 2024), meaning participants who have more AUD symptoms (in full-sample analyses) *may* have lower levels of anti-inflammatory cytokines. Second, this finding is in contrast to evidence that elevated cytokine levels can differentiate AUD status, although those findings are from AUD patients in residential treatment, where AUD is likely to be particularly severe (Leclercq et al., 2012). Finally, it is possible that these were spurious associations in the current study.

### 4.1 Limitations & future directions

While the co-twin control analyses applied here are rigorous and suggested familial confounds explain associations between heavier alcohol use and dysregulated cytokine levels in healthy adults, future studies can build on this work in important ways. As noted, participants had low rates of AUD, and it is possible that alcohol use was below any threshold that would increase inflammation. Thus, similar family-controlled or other quasi-experimental studies with some proportion who have severe AUD would be informative. Additionally, the current sample was healthy and had few health problems. However, it is well known that psychiatric disorders (including AUD and depression) co-occur with other health problems (Hagerty et al., 2019), and it is possible that the relationship between cytokines and AUD and depression is better explained by broader health problems and/or confounding behaviors (e.g., sleep). Finally, a notable limitation was the cross-sectional nature of this analysis, which highlights the need for longitudinal/prospective data to examine the timing of AUD onset, MDD onset, and elevated cytokines.

## 5 Conclusions

The current study analyzed biomarker and clinical data from over 900 community adults to examine whether elevated cytokine levels are associated with AUD and depression and whether familial confounds may explain these effects. We did not find significant associations between having more AUD or depression symptoms and elevated cytokine levels. Thus, our findings suggest that cytokine levels are unrelated to AUD symptoms, low levels of alcohol, and MDD symptoms in an otherwise healthy sample of middle-aged adults.

## CRediT authorship contribution statement

**Ryan Bruellman:** Conceptualization, Formal analysis, Methodology, Software, Visualization, Writing – original draft, Writing – review & editing. **Chandra A. Reynolds:** Data curation, Writing – review & editing. **Andrew Smolen:** Data curation, Investigation, Resources, Writing – review & editing. **Donald Evans:** Data curation, Investigation, Resources, Writing – review & editing. **Jarrod M. Ellingson:** Conceptualization, Formal analysis, Methodology, Supervision, Writing – original draft, Writing – review & editing.

## Declaration of competing interest

The authors declare that they have no known competing financial interests or personal relationships that could have appeared to influence the work reported in this paper.

## Data Availability

For inquiries about data availability from the current study please contact the corresponding author and study PIs. The data contain potentially identifying or sensitive information, and sharing may be restricted according to participant or IRB directives. Requests for data will require the completion of a data use agreement, documentation of training in the protection of human subjects, and purpose of use.

## Appendix A Supplementary data

Supplemental Figures: https://osf.io/mafkt/?view_only=f104ba4f27374d39867bbe9dc8b1e7cf.
