# Supplementary material for: Inflammation, mental health, and alcohol behaviors: Testing links leveraging a familial community sample
Source: Brain Behav Immun Health. 2026 Mar 26;53:101229. doi: 10.1016/j.bbih.2026.101229 (PMC13066790; doi:10.1016/j.bbih.2026.101229)
Supplement: Multimedia component 1 [file mmc1.pdf]

MDD Outcome Cytokine Model

MDD = outcome

	Diet factors				Univ	Demographic									Sensitivity											
	Fruit/Veg	Fiber	Calcium	Sugar	Cytokine	Cytokine	Male	Age	Fruit/Veg	Fiber	Calcium	Sugar	BMI	DepMed	Cytokine	Male	Age	Fruit/Veg	Fiber	Calcium	Sugar	BMI	DepMed	Non-white	Hispanic	Batch
**Pro-Inf**			*	*										*									*			
**IL-1b**			*											*									*			
**IL6**			*											*									*			*
**TNFα**					*	*								*									*			
**IFN-γ**														*									*			
**IL-4**	*													*									*			*
**IL-8**														*									*			
**IL-10**				*										*									*			
**IL-12**					*	*								*									*			
**CRP**			*											*									*			*
**IL-5**				*										*									*			
**IL-22**				*										*									*			
