# Supplementary material for: Inflammation, mental health, and alcohol behaviors: Testing links leveraging a familial community sample
Source: Brain Behav Immun Health. 2026 Mar 26;53:101229. doi: 10.1016/j.bbih.2026.101229 (PMC13066790; doi:10.1016/j.bbih.2026.101229)
Supplement: Multimedia component 3 [file mmc3.pdf]

Alcohol Frequency Models (Alc F)

	Diet factors				Univ	Demographic									Sensitivity											
	Fruit/Veg	Fiber	Calcium	Sugar	Alcohol Freq	Alc F	Male	Age	Fruit/Veg	Fiber	Calcium	Sugar	BMI	DepMed	Alc F	Male	Age	Fruit/Veg	Fiber	Calcium	Sugar	BMI	DepMed	Non-white	Hispanic	Batch
**Pro-Inf**			*	*				*					*									*				*
**IL-1b**			*		*	*		*							*											*
**IL6**			*					*					*									*				*
**TNFα**								*					*				*					*				*
**IFN-γ**																										*
**IL-4**	*				*	*									*										*	*
**IL-8**								*					*				*					*				*
**IL-10**				*				*				*	*								*	*				
**IL-12**								*					*									*				*
**CRP**			*				*						*	*		*						*	*			
**IL-5**				*									*									*				*
**IL-22**				*	*	*									*											
