# Supplementary material for: Inflammation, mental health, and alcohol behaviors: Testing links leveraging a familial community sample
Source: Brain Behav Immun Health. 2026 Mar 26;53:101229. doi: 10.1016/j.bbih.2026.101229 (PMC13066790; doi:10.1016/j.bbih.2026.101229)
Supplement: Multimedia component 6 [file mmc6.pdf]

ICCs	Outcome												
	MDD	Pro-inf	IL-1b	IL-6	TNFα	IFN-γ	IL-4	IL-8	IL-10	IL-12	CRP	IL-5	IL-22
**Adoptees**	<0.01	<0.01	<0.01	<0.01	<0.01	<0.01	<0.01	<0.01	0.01	<0.01	0.04	0.02	0.07
**Biological Siblings**	0.13	0.18	0.27	<0.01	0.33	<0.01	0.35	<0.01	<0.01	<0.01	0.21	0.18	<0.01
**DZs**	<0.01	0.32	0.27	0.27	0.37	0.21	0.34	<0.01	0.18	0.30	0.29	0.42	0.01
**MZs**	0.20	0.49	0.54	0.27	0.53	0.42	0.43	0.33	0.23	0.39	0.61	0.28	<0.01

Intraclass correlation coefficients (ICCs) are calculated from the univariate model linear mixed-effect result; DZs = dizygotic twins; MZs = monozygotic twins
