# Supplementary material for: Inflammation, mental health, and alcohol behaviors: Testing links leveraging a familial community sample
Source: Brain Behav Immun Health. 2026 Mar 26;53:101229. doi: 10.1016/j.bbih.2026.101229 (PMC13066790; doi:10.1016/j.bbih.2026.101229)
Supplement: Multimedia component 7 [file mmc7.pdf]

Pro-Inf	Estimate	SE	DF	Pr > |t|	Lower	Upper
Intercept	0.083	0.052	204	0.114	-0.020	0.185
AUD Between Effect	-0.189	0.132	205	0.155	-0.450	0.072
MZ AUD Between	-0.020	0.185	205	0.914	-0.385	0.345
AUD Within Effect	-0.171	0.136	205	0.209	-0.439	0.096
MZ AUD Within	0.047	0.176	205	0.791	-0.300	0.392
Age	0.003	0.039	205	0.942	-0.073	0.079
Sex	0.052	0.105	205	0.619	-0.155	0.260

IL-1b	Estimate	SE	DF	Pr > |t|	Lower	Upper
Intercept	0.067	0.055	204	0.227	-0.042	0.176
AUD Between Effect	-0.183	0.137	205	0.182	-0.454	0.087
MZ AUD Between	-0.022	0.197	205	0.910	-0.411	0.366
AUD Within Effect	-0.231	0.149	205	0.123	-0.524	0.063
MZ AUD Within	0.159	0.187	205	0.397	-0.210	0.529
Age	0.000	0.041	205	0.999	-0.081	0.081
Sex	-0.001	0.112	205	0.996	-0.221	0.220

IL-12	Estimate	SE	DF	Pr > |t|	Lower	Upper
Intercept	0.094	0.055	204	0.089	-0.014	0.202
AUD Between Effect	-0.243	0.142	205	0.089	-0.523	0.037
MZ AUD Between	0.190	0.195	205	0.331	-0.194	0.575
AUD Within Effect	-0.241	0.153	205	0.116	-0.542	0.060
MZ AUD Within	0.055	0.201	205	0.785	-0.341	0.451
Age	0.053	0.041	205	0.199	-0.028	0.134
Sex	0.055	0.111	205	0.622	-0.164	0.273

IL-10	Estimate	SE	DF	Pr > |t|	Lower	Upper
Intercept	0.120	0.052	204	0.023	0.017	0.223
AUD Between Effect	-0.086	0.133	205	0.521	-0.348	0.177
MZ AUD Between	-0.118	0.186	205	0.526	-0.484	0.248
AUD Within Effect	-0.104	0.150	205	0.488	-0.400	0.191
MZ AUD Within	0.005	0.207	205	0.980	-0.403	0.414
Age	-0.001	0.039	205	0.985	-0.078	0.077
Sex	0.014	0.105	205	0.891	-0.194	0.222

IL-4	Estimate	SE	DF	Pr > |t|	Lower	Upper
Intercept	0.071	0.059	204	0.228	-0.045	0.187
AUD Between Effect	-0.105	0.147	205	0.475	-0.400	0.185
MZ AUD Between	-0.100	0.209	205	0.633	-0.511	0.311
AUD Within Effect	-0.157	0.150	205	0.299	-0.453	0.140
MZ AUD Within	0.023	0.202	205	0.910	-0.375	0.421
Age	-0.015	0.043	205	0.737	-0.100	0.071
Sex	0.145	0.118	205	0.224	-0.089	0.378

Supplemental Figure 6 - Co-twin control analysis results for selected cytokine outcomes and alcohol use disorder. AUD = Alcohol use disorder; Pro-Inf = pro-inflammatory; SE = Standard error; DF = Degrees of freedom; MZ = Monozygotic twins
